# Food prices and the wages of the poor: A cost-effective addition to high-frequency food security monitoring

**DOI:** 10.1016/j.foodpol.2024.102630

**Published:** 2024-05

**Authors:** Derek Headey, Fantu Bachewe, Quinn Marshall, Kalyani Raghunathan, Kristi Mahrt

**Affiliations:** The International Food Policy Research Institute, United States

**Keywords:** Food prices, Food crises, Food security, Nutrition, Wages, Healthy diets, Early warning systems

## Abstract

The affordability of nutritious food for “all people, at all times” is a critically important dimension of food security. Yet surprisingly, timely high-frequency indicators of food affordability are rarely collected in any systematic fashion despite price volatility emerging as major source of food insecurity in the 21st Century. The 2008 global food crisis prompted international agencies to invest heavily in monitoring domestic food prices in low and middle income countries (LMICs). However, food price monitoring is not sufficient for measuring changes in diet affordability; for that, one must also measure changes either in income or in an income proxy. We propose using the wages of unskilled workers as a cheap and sufficiently accurate income proxy, especially for the urban and rural non-farm poor. We first outline alternative measures of “food wage” indices, defined as wages deflated either by consumer food price indices or novel healthy diet cost indices. We then discuss the conceptual strengths and limitations of food wages. Finally, we examine patterns and trends in different types of real food wage series during well-known food price crises in Ethiopia (2008, 2011 and 2022), Sri Lanka (2022) and Myanmar (2022). In all these instances, food wages declined by 20–30%, often in the space of a few months. In Myanmar, the decline in real wages during 2022 closely matches declines in household disposable income. We strongly advocate tracking the wages of the poor as a timely, accurate and cost-effective means of monitoring food affordability for important segments of the world’s poor.

## Highlights

• High-frequency indicators of food affordability are unavailable in LMICs.
• Agencies invested in food price data after 2008, but not income or wage data.
• We propose real wages of unskilled workers as a cheap and accurate income proxy.
• We show that real food wages declined by 20–30 % in well-known food crises in 2022.
• We outline strengths, limitations and scope for improved measurement of real wages.

## 1 Introduction

The most widely used definition of food security refers to ensuring the affordability of nutritious food for “all people, at all times” (FAO, 1996). However, timely accurate indicators of food security are difficult to collect in a cost-effective manner, especially during rapidly evolving crises when in-person surveys are both costly and relatively slow to yield results, or even infeasible due to conflict or other logistical barriers. In a world increasingly exposed to extreme weather events, major conflicts, and economic volatility, it is surely advisable for governments and development partners in LMICs to invest much more in high-frequency household surveys that measure food security and other key welfare measures (Headey and Barrett, 2015). Until they do so, however, it is critically important to find other means of improving food and nutrition security monitoring.

**Fig. 1:**
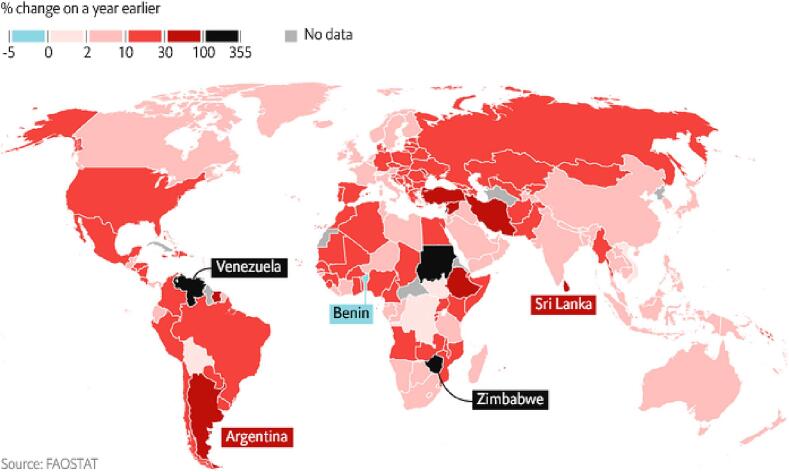
Food inflation in 2022: percentage change on a year earlier) and map presented by .

Source: Underlying data from FAOSTAT ([Bibr b0140][Bibr b0325]

One major source of food and nutrition insecurity in the 21st century has been food price volatility. After decades of stagnation, international food prices spiked sharply in what became widely known as the 2008 global food crisis (Headey and Fan, 2010, IFPRI, 2008; The World Bank, 2008). Prices spiked again in 2010–11, however, and then more recently in 2021–2022 in the wake of COVID-19 and Russia’s invasion of Ukraine (Abay et al., 2023). In 2022, year-on-year food inflation reached 76 % at the peak of Sri Lanka’s financial crisis, 60 % in war-torn Myanmar, and 40 % in Pakistan where a macroeconomic crisis coincided with unprecedented floods, while countries like Sudan, Zimbabwe and Venezuela suffered from hyperinflation (Fig. 1). Given that some 3 billion people could not afford a healthy diet even prior to this recent global food crisis (FAO, IFAD, UNICEF, WFP, and WHO, 2020), we should be deeply concerned about the consequences of food price crises for poverty and food insecurity (Arndt et al., 2023), as well as malnutrition (Osendarp et al., 2022, Headey and Ruel, 2023).

In the wake of 2008 food crisis, one perceived failure on the part of the development community at large was inadequate national food price monitoring to inform early warning systems and provide timely data on the potential welfare impacts of rising food prices. Subsequently, significant efforts were made to improve the monitoring of food prices and to integrate them into multidimensional food security surveillance and early warning systems. The most prominent examples include the Food and Agriculture Organisation (FAO) *Global Information and Early Warning System on Food and Agriculture*, the USAID *Famine Early Warning System*, and the World Food Programme’s (WFP) *Market Monitor* (among others.^1^

But while efforts to collect food price data are certainly necessary for more effective food and nutrition security surveillance, they are not sufficient. It is changes in the *affordability* of diet baskets that matter for food and nutrition security, not changes in their nominal cost, since incomes, too, can change quickly for better or worse, depending on the economic situation at hand.

Our innovation in this paper is two-fold: first, we propose going beyond conventional food price indices to additionally measure indices that capture the cost of hypothetical healthy diets derived from nutrition recommendations. Second, we argue that the wages of various types of unskilled workers are a good income proxy for important segments of poor and vulnerable populations in LMICs, especially the urban and rural non-farm poor. By measuring wage changes relative to food price changes one can derive a variety of measures of the real wages of the poor, which can then be used as high-frequency and cost-effective food insecurity indicators. Our study therefore links both to the more general literature on food security measurement (Barrett, 2010, Headey and Ecker, 2013, Jones et al., 2013, Leroy et al., 2015), and to a more specific and more recent literature on measuring the affordability of healthy diets (Dizon and Herforth, 2018, Dizon et al., 2019, Mahrt et al., 2019, Raghunathan et al., 2021, Hirvonen et al., 2020).

These studies on the affordability of healthy diets have become influential in recent years, with the UN’s *State of Food Insecurity and Nutrition in the World* (SOFI) annual reports (FAO, IFAD, UNICEF, WFP, and WHO, 2022) mainstreaming the cost and affordability of healthy diets in UN food security reporting. But while this recent focus on healthy diet affordability is welcome, the global reporting in the SOFI reports is limited by low-frequency measurement of both food prices and household incomes or expenditures, and only a few LMICs have started to consider high frequency reporting of diet affordability.^2^ Acknowledging this, the FAO has stated:

*“FAO is focusing attention on the pursuit of healthy diets and transformations of food systems to ensure healthy diets are affordable for all, all year round. FAO is encouraging Governments to put the affordability of healthy diets at the centre of their agricultural policies, social protection, and investment decisions.”*^3^

In this paper we contend that collecting wages for unskilled workers – or employing existing wage data, if available – will provide a relatively accurate and low-cost approximation of the income trends of those economic groups most vulnerable to higher food prices, namely the non-farm poor. We acknowledge that wages may not be a good proxy for the incomes of households primarily dependent on farming, many of whom are very poor and food-insecure, and propose that real food wages be thought of as a complement to more agriculture-oriented food security indicators measured at high-frequency, such as various climate-based metrics in early warning systems (Backer and Billing, 2021, Funk et al., 2019, Rembold et al., 2019).

We first outline alternative methods for constructing alternative real wage indicators (Section 2), before discussing the conceptual strengths and limitations of the real wages of the poor as a food security indicator (Section 3). We then examine whether high-frequency indicators of food wages are indeed sensitive to major economic and political shocks using real-world examples from Ethiopia, Sri Lanka and Myanmar (Section 4). Section 5 concludes by summarizing why we contend that monitoring of food wages could be a feasible, cheap and accurate tool for providing almost real-time monitoring of food security for large segments of the world’s poor and vulnerable population.

## 2 Alternative methods for constructing real food wage indicators

The measurement of “food wages” is almost as old as economics itself, with early economists (Malthus, 1798, Playfair, 1821, Smith, (1776).) comparing wages of working-class households to the price of bread or other grains.^4^ Modern development economists have often focused on “rice wages” in Asia because of because of the overwhelming importance of rice in the diet of poor agricultural workers in countries like Bangladesh and Indonesia (Azam, 1993, Mazumdar and Sawit, 1986, Zhang et al., 2014). But real wage monitoring has rarely been used for food security measurement in any systematic fashion. In this section we therefore lay out alternative food price indices and wage indicators, and discuss the measurement of real wage levels and trends.

### 2.1 Alternative food price indices

In this study we consider a range of different types of wage series for less skilled or semi-skilled occupations, as well as three different types of food price indices:

(1) a standard consumer food price index (FCPI);
(2) a poor person’s food price index (PP_FCPI); and
(3) a cost of healthy diet price index (COHD).

The standard *consumer food price index* weighs the prices of different foods in a country by expenditure shares of that country’s consumers and keeps the weights fixed over time (with occasional updates). The *poor person’s food price index* is very similar but derives weights from the expenditure shares of the poorest households (Bachewe and Headey, 2017). The *cost of healthy diet price index* is instead based on the cheapest possible combination of foods that satisfies quantitative food group intake recommendations in national food-based dietary guidelines. The implicit weights in this index are the food group quantities recommended by nutritionists, while the cheapest price within each food group acts as a second layer of weighing different foods.

There are advantages and disadvantages to each of these indicators. Consumer food price indices reflect price changes for foods purchased by the average citizen in a country (or the average poor citizen in the case of PP_FCPI), capturing price changes for the foods people actually eat. However, costing a recommended healthy diet – whilst not reflecting existing consumption patterns – is nutritionally meaningful because healthy diets satisfy requirements for complete nutrition (Herforth et al., 2019). This distinction is conceptually important because in most LMICs the diet of an average person – and certainly of a poor person – will generally be inadequate in many nutrients because of excessive consumption of starchy staples and under-consumption of nutrient-dense foods (Mahrt et al., 2022). Moreover, while conventional food price indices have constant weights that do not allow for substitution across seasons or years, the COHD selects the cheapest food item in each food group at each time point, so does allow for substitution based on market prices. In summary, for a more nutrition-oriented perspective of food security, the COHD index is more relevant, while the poor person’s price index is more relevant from a traditional calorie-oriented food security perspective.

In addition to conceptual differences, there may be empirical instances where conventional price indices and healthy diet price indices show different trends, because the healthy diet index gives greater weight to nutrient-dense foods but also selects least-cost foods. Relative to staple foods, nutrient-dense foods are more perishable and less internationally traded, so their price movements could be quite different to staples. Fruits and vegetables exhibit much more seasonality than staple foods, for example (Bai et al., 2020, Raghunathan et al., 2021). Moreover, different types of shocks may affect commodities differently; e.g. international price increases should have greater effects on tradable non-perishable foods relatively to fruits and vegetables, unless key inputs are also tradable.^5^ Hence, we suggest that it may often be insightful to use both conventional and healthy diet food price indices side-by-side.

### 2.2 Alternative wage indicators for lower skilled occupations

While a large share of any LMIC workforce is engaged in some kind of labor, specific activities differ in function and skill level, as well as the degree of informality. In agricultural areas it is common – especially in Asia – to hire both men and women to perform a variety of functions such as planting, weeding and harvesting, though male wages are generally much higher than women’s wages (Raghunathan et al., 2021). In much of Asia, agricultural workers are landless or only hold very small landholdings, and wages are low, making them the poorest of the poor (Castaneda et al., 2016, Headey et al., 2010). Outside of agriculture, daily/casual laborers can be hired for a variety of tasks, especially construction, and many laborers may work seasonally in both agricultural and non-agricultural activities. However, in severe economic crises it may also be important to consider wages for occupations not normally associated with extreme poverty – such as semi-skilled construction workers, shop assistants or tradesmen – since these households are vulnerable to falling into poverty during more extreme economic crises, as we show in the Sri Lankan example below.

Additional complexities pertain to data collection and access. Wage data are collected in most household surveys and labor force surveys, but high frequency measurement (such as monthly) is by no means universal in LMICs, particularly in Africa. Even when wage data are collected regularly, they may not be disseminated publicly or on a timely basis. Wages may also be collected by different institutions even in the same country: at least three different government agencies collect wage data in India, two in rural areas and one in urban areas. In many countries the WFP also collects wage data in conjunction with primary food price surveys. During the COVID-19 crisis in Myanmar, IFPRI also started collecting food price and wage data through phone surveys of food vendors (MAPSA, 2022a).

### 2.3 Estimating patterns and trends in the real food wages of the poor

Estimating patterns and trends in food wages for any given labor activity involves the straightforward deflation of a nominal wage series by a food price deflator, such as the food CPI, poor person’s food CPI, or the COHD.^6^ Specifically, the real wage index ($w^{r}$) at location *I* and time *t* is calculated by dividing the nominal wage (*w^n^*) by the price index (*P*):

(1) $w_{i,t}^{r}=\frac{w_{i,t}^{n}}{P_{i,t}}$

When the price index (*P*) is the cost of healthy diet, it should also be noted that $w^{r}$ has a very intuitive interpretation, since it measures the number of healthy diet days that can be purchased with the daily wage if $w^{r}>1$, or the fraction of healthy diet days if $w^{r}<1$. Note that $w^{r}$ can always be calculated separately for working-age men and women if sex-specific wages or dietary recommendations are available, with the obvious utility in identifying gender differences in real wages across locations and time.

As an alternative to the real wage in (1), it is also possible to calculate the inverse ratio to measure the wage-based affordability of a healthy diet (A) as the healthy diet’s cost relative to the wage rate:

(2) $A_{i,t}=\frac{P_{i,t}}{w_{i,t}^{n}}$

If $P<w^{n}$, then a daily wage suffices to buy at least one healthy diet day for one individual, excluding non-food expenditure requirements.^7^ However, it would also be possible to calculate healthy diet costs for hypothetical family sizes to factor in a given number of children who are assumed not to earn an income and to have lower calorie requirements than an adult. This approach is often used by the WFP in the context of nutrient-adequate diet cost and affordability calculations (*Fill the Nutrient Gap*) for social protection programming (WFP, 2020).

While the real wage rate in equation (1) is insightful for comparing wages across different economic activities, sexes and locations (depending on the data), it is the change in the real wage that is useful in assessing high-frequency changes in food insecurity. Percentage changes in real wages over time approximate to the percentage change in the nominal wage minus the percentage change in the price index:

(3) ${\dot{w}}_{i,t}^{r}={\dot{w}}_{i,t}^{n}-{\dot{P}}_{i,t}$

If the nominal wage is unchanged when food prices increase, then the percentage reduction in the real wage is equal to the percentage increase in the food price index. In many circumstances we would expect some nominal wage rigidity during very sudden food price spikes, as labor market adjustments to higher food prices take time, although in economies that are experiencing rapid economic or also just “overheating” it is quite possible that food inflation is accompanied by significant wage inflation. We demonstrate this with the Ethiopian example in Section 4. We also note that there may also be a divergence between rural wages and urban wages when food prices increase, especially if higher farmgate prices incentivize farmers to hire more rural labor (Jacoby, 2016).

## 3 Potential strengths and limitations of real wages of the poor as food security indicators

The key rationale for analyzing real wages for unskilled activities is that changes in real wages will be predictive of disposable income trends for large sections of the poor. In this section we examine that rationale in more detail, considering both theoretical arguments and empirical evidence.

### 3.1 Are real wages for unskilled labor activities predictive of poverty and food insecurity?

In economics, the rationales for measuring real wages of unskilled workers are both theoretical and empirical. Angus Deaton and Jean Dreze (2002), who used state-level trends in wages of unskilled labor to re-assess official poverty trends in India, state the case eloquently:

“real wages can be used to provide some information about other poverty indexes … it is also possible to think about the real wage as a rough poverty indicator in its own right. The idea is that, if the labour market is competitive … then the real wage measures the ‘reservation wage’, i.e., the lowest wage at which labourers are prepared to work. This has direct evidential value as an indication of the deprived circumstances in which people live (the more desperate people are, the lower the reservation wage), independently of the indirect evidential value arising from the statistical association between real wages and standard poverty indexes such as the headcount ratio.” Source: Deaton and Dreze (2002), page 3737

One concern with this argument is that the association between wage dependency and poverty might be stronger in Asia, where there are higher rates of landlessness, greater use of hired labor in intensive agricultural systems, and more poor people who rely on selling their labor for cash in both rural and urban areas. Are wages likely to be just as predictive of the incomes of the poor in sub-Saharan Africa, where there are often exceptionally high rates of food insecurity? We think yes, for two reasons, albeit mainly for households not primarily dependent on working on their own farm.

First, Africa is becoming increasingly urbanized, and it is precisely the urban poor in Africa who are more likely to report greater food insecurity in the context of rising food prices, since they purchase most of the food they consume (Verpoorten et al., 2013). Hence, in terms of vulnerability to food price shocks, real food wages are highly relevant to Africa’s non-farm poor.

Second, wages are an increasingly important source of income for African households as their economies undergo economic transformation and demographic pressures in rural areas. A recent study found that wage employment accounted for 32 % of total hours worked in six African countries,^8^ with own-farm and non-farm enterprise work each accounting for 34 % (Fox and Gandhi, 2021). Among urban populations in these six countries, wage work accounted for 51 % of hours worked in urban areas compared to 30 % for non-farm self-employment (Fox and Gandhi, 2021). Studies also show that wage employment is also increasing over time, even in low-income countries experiencing sluggish economic growth. In Malawi, for example, one study found that the average working age adult worked just 116 h per year in casual labor in 2010, but almost 300 h per year by 2019, compared to just 142 h in own-farm labor (Van Capellen and De Weerdt, 2023).

To further assess how strongly wages are correlated with the incomes of the poor at a global level – and not just in Asia – we combine (1) World Bank (2022a) estimates of the average incomes of the poorest 40 % of the population in a given country in 2011, and (2) wages, service charges and salaries from various activities of different skill levels sourced from the 2011 International Comparison Program (World Bank, 2013). Table 1 reports correlations between the incomes of the poorest 40 % and wage/salary rates.

**Table 1:** Cross-country correlations between the average income of the poorest 40% of a population and wages/salaries for occupations of differing degrees of skill, formality and manual labor.

**Less skilled, less formal, more manual occupations**			**More skilled, more formal, less manual occupations**		
	No. of countries	Correlation (*=p < 0.01)		No. of countries	Correlation (*=p < 0.01)
Servant’s wage	64	0.73*	Prison guard salary	60	0.36*
Construction wage	75	0.64*	Police salary	58	0.28
Garage service charge	59	0.82*	Auxiliary nurse salary	67	0.44*
Bricklayer wage	75	0.61*	Primary teacher salary	61	0.36*
Plumber wage	75	0.55*	Secretary salary	62	0.15
Carpenter wage	74	0.57*	Bookkeeper salary	50	0.35
Steelworker wage	70	0.62*	Computer operator salary	63	0.11
Electrician wage	74	0.50*	Public health official salary	62	0.15

Source: Authors’ analysis from income estimates from the World Bank’s (2022a) Poverty and Inequality Platform and wage and salary data from the 2011 International Comparison Program database (World Bank, 2013). Both wages and incomes are expressed in 2011 purchasing power parity dollars.

What do we find? First, the average incomes of the poorest 40 % are strongly and significantly correlated with wage rates of servants (0.73) and low-skilled construction labor (0.64), as well as other semi-skilled construction sector activities. In contrast, correlations between wages/salaries for more skilled activities and occupations have much weaker and often statistically insignificant associations with the income levels of poor people, as one would expect.

### 3.2 Potential limitations of real food wages as a food security indicator

There are several potential limitations to using real wages to monitor food insecurity at high frequency.

The first is a very important limitation: real wages are unlikely to be a good proxy for disposable incomes or food security for households that are highly dependent on farming, and such households are often food insecure. In that context, more agriculture- or climate-focused predictors of food security are needed, and indeed climate-based early warning systems have generally been shown to be relatively good predictors of food crises in rural Africa (Fritz et al., 2019, Funk et al., 2019). Moreover, when food prices increase, it is also possible that farmers benefit from higher incomes even as non-farm households experience declining disposable incomes. So, both agriculture-focused and wage-focused indicators will be insufficient by themselves; indeed, they are complements not substitutes.

A second potential limitation is that many non-farm poor earn income from self-employment rather than wage employment (Fox and Gandhi, 2021, Castaneda et al., 2016, Robles Aguilar and Sumner, 2020). Theoretically, however, there are logical reasons to expect quite a strong correlation between self-employment incomes and wages because, for the poor especially, both types of work tend to be informal, low skill and therefore low entry. Potentially, casual labor and self-employment activities are integrated within a common labor market for low skilled labor since a poor person might easily switch from casual labor to low-entry forms of self-employment (e.g., street vendors, rickshaw drivers), and vice versa. In Section 4 we show this to be the case for a high-frequency panel survey in Myanmar conducted during its economic crisis in 2022: real wages and the incomes of households dependent on self-employment declined by similar margins during a period of exceptionally high food inflation (Fig. 5).

A third limitation of real wage trends Is that in economic crises they may not accurately reflect reductions in wage *incomes* because of reductions in hours worked due to falling demand for labor. The size of this bias is difficult to assess. In the Myanmar example presented below we find that trends in real wages correspond closely to trends in income for wage-dependent households, suggesting little or no bias. One explanation may be high rates of migration in response to severe economic crises, which leads to a tightening of labor markets as workers move from labor-deficit to labor-surplus regions, including neighboring countries. A study in one of our focus countries, Myanmar, found that approximately 3.6 million individuals moved between December 2021 and June 2022, or approximately 10 % of the total working age population (MAPSA, 2023). In another of our focus countries, Sri Lanka, official emigration from January 2022 to March 2023 was 370,000 or 4.2 % of the working age population, with unofficial migration likely much higher (The Economist, 2023). Even so, it is indeed likely that real wages and real wage incomes could diverge in mean circumstances because of changes in hours worked. One solution is for wage surveys to collect estimates of “normal” daily hours of work in addition to wage rates, as has been done in India in the past (Raghunathan et al., 2021).

A fourth limitation is that some labor markets in LMICs may still operate with in-kind wages (food- and/or boarding-in-kind), especially in rural areas and especially among women. To investigate this issue, albeit only for women, we use data from the Demographic Health Surveys (ICF International, 2022) for almost 400,000 women in 62 LMICs, which asks female respondents about whether they were paid in cash, in-kind, or both cash and in-kind. The results, reported in Appendix Table A1 show that wages-in-kind are much less common than cash-only payments in most regions, with some exceptions, such as rural Uganda. In Appendix Table A2 we look at forms of payment for working women in sub-Saharan Africa by occupation and find that in-kind payments are only common for women working in agriculture. These data therefore suggest that wage data in the agricultural sector in Africa and a few other LMICs could still be distorted by in-kind payments. A potential solution is to specify wage quotations for daily labor without in-kind payments if that form of payment exists, but it is also advisable for agencies to assess how common in-kind wages are in a given locality prior to collecting or analyzing wage data.

A fifth issue to consider is the representativeness of food and wage price data. Unlike household surveys, where methods for achieving representativeness are quite standardized, the representativeness of price and wage surveys are not always so transparently described. Government consumer price surveys – used for official inflation estimates – sometimes underrepresent rural locations and the food vendors they use because it is relatively costly to conduct in-person surveys in remote areas (ILO, IMF, OECD, EU, UN, and World Bank, 2020). However, since households in more remote rural areas tend to have greater dependence on own-farm income rather than wage income, this bias may not be so problematic; nevertheless, potential urban bias or food vendor bias should be assessed. Moreover, if wage and price data are from different sources, then attention needs to be the representativeness and comparability of price and wage data.

## 4 Food wages in food crises: are they sensitive to shocks and predictive of food insecurity?

The most important test of our claim that food wages are a useful high-frequency indicator of food security is that food wages should be sensitive to food price shocks. In this section we present evidence from food crises in Ethiopia, Sri Lanka, and Myanmar. In each case we use somewhat different types of wage and food price series, and very different data sources, to demonstrate that a range of alternative real wage estimates are possible from both primary and secondary data sources.

In Ethiopia we use secondary data sources but conduct relatively intricate calculations of a poor person’s food CPI using household expenditure data on consumption patterns of the urban poor in each region of Ethiopia, following Bachewe and Headey (2017). The Ethiopian data is unique in covering two decades (2002–2022) in which there were three separate food crises, but also periods of rapid economic growth and relative food price stability. In Sri Lanka, we look at wages for unskilled and semi-skilled occupations in 2021 and 2022, both deflated against a standard food CPI, and both sourced from published bulletins from the Central Bank of Sri Lanka. Finally, in Myanmar we use wages of both agricultural and construction laborers in rural and urban areas, respectively and a cost of healthy diet food price index, the underlying data for which come from a high-frequency household phone survey conducted by IFPRI. The advantage of the household phone survey in this context is that we can also compare real wage trends to household income and poverty trends in rural and urban areas, and for different household livelihood types.

### 4.1 Trends in the poor person’s consumer food price index and real food wages during multiple food price crises in Ethiopia and periods of rapid economic growth: 2002–2022

Progress against poverty and food insecurity in Ethiopia has been stop-start, and at times paradoxical. The country’s aggregate economic performance, as measured by GDP growth, has been extremely successful, with economic growth rates ranging between 6 and 14 % in every year between 2004 and 2020, only dipping below 6 % after the civil war broke out in late 2020. However, despite high growth, acute food insecurity challenges were still common in this period, sometimes due to drought in rural areas, but also due to major food price spikes. In 2007–2008 Ethiopia had one of the highest food inflation rates in the world, driven by international price increases, macroeconomic overheating, and local crop failures (Durevall et al., 2013). Survey-based research published just after the 2008 food price crisis concluded that the urban poor were hard hit by food inflation, while impacts in rural areas were more nuanced (Hadley et al., 2012, Kumar and Quisumbing, 2013, Ticci, 2011), with farmers likely benefiting in net terms (World Bank, 2015). Food price inflation peaked again in a second crisis in 2010–11. In the years that followed, prices of some commodities, particularly animal-sourced foods (Bachewe et al., 2017), continued to increase more secularly, before food prices again rose sharply in from 2020 to 2022 due to a combination of civil war, domestic macroeconomic problems, and high international food prices.

In an earlier study, Bachewe and Headey (2017) used the Ethiopian government’s *Consumer Price Survey* to gauge the impact of the 2007–08 and 2010–11 food price crises on the real wages of the urban poor. This survey covers 120 market locations in urban Ethiopia (including small urban towns with as few as 5000 people) for prices of food and non-food goods and services. One of the non-food services priced in this survey is the wage rate for daily laborers, largely working in construction. To analyze real wages of the urban poor, Bachewe and Headey (2017) constructed region-specific poor person’s food price indices based on the consumption patterns of the bottom 40 % of the expenditure distribution. They then use this poor person’s food price index to deflate nominal wages to measure the real wages of the poor (food wages) over 2001–2012. In this study we replicate that analysis for 2001–2022.

**Fig. 2:**
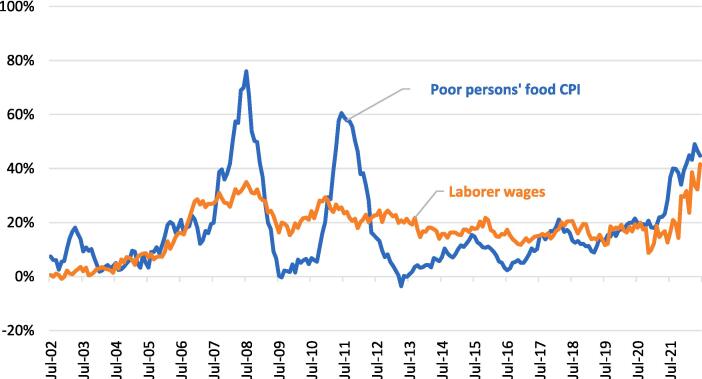
Rolling 12-month inflation (%) in the poor person’s food CPI and daily laborer wages in Ethiopia, averaged across 120 locations in urban Ethiopia from June 2002 to June 2022). The poor person’s food price index weights each food item by regional consumption shares for the urban poor, as described in .

Source: Authors’ analysis using ESS retail price data ([Bibr b0125][Bibr b0040]

Before turning to real wages, it is useful to examine the dynamics of food prices and wages separately. In Fig. 2 we therefore report rolling 12-month inflation rates for both the poor person’s food CPI (blue line) and the daily laborer wage rate (orange line) over the full period from July 2002 to June 2022. As we discussed in the context of equation (3), changes in the real wage are simply a race between changes in the nominal wage and changes in the food price index, such that whenever the blue food inflation line exceeds the orange wage inflation line one can infer that real wages are declining, and conversely that real wages are increasing if the orange line exceeds the blue line in Fig. 2.

Given this, Fig. 2 illustrates that the food crises of 2007–08 and 2010–11 were indeed periods in which food inflation quickly outpaced wage inflation, and by large margins. Food inflation for the urban poor peaked at almost 80 % in mid-2008 and at 60 % in mid-2011. However, nominal wage rates did not stand still either. In fact, until December 2007, wage inflation largely kept pace with food inflation, implying no major secular change in the real wages of the poor. Then in early 2008 food inflation started to far outpace wage inflation, though wage inflation itself was still very high, reaching 33 % in July 2008 when food inflation also peaked. Indeed, wage inflation was high throughout this period, averaging 24 % from September 2006 to September 2013. Moreover, after the 2011 price spike, wage inflation actually outpaced food inflation in every month from the five years from September 2012 to September 2017, and often by significant margins, implying significant growth in real wages. It was only from January 2021 – when the civil war escalated and international prices started rising – that food inflation once again outpaced wage inflation. Even so, wage inflation reached 40 % in June 2022 at the end of our sample, just below the 47 % food inflation rate observed at the same time.

**Fig. 3:**
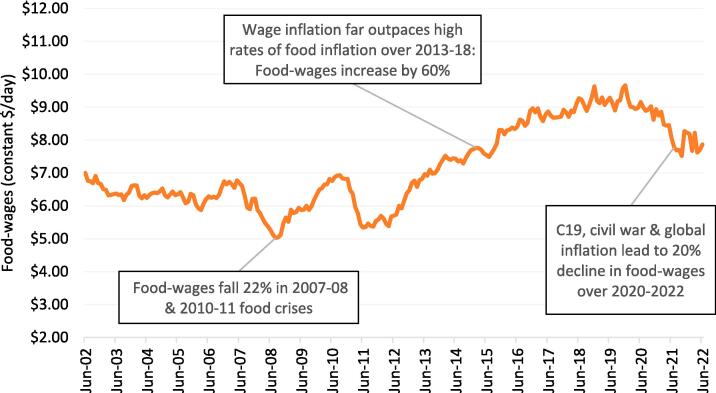
Trends in monthly “food wages” for daily laborers, averaged across 120 locations in urban Ethiopia from June 2002 to June 2022 (2011 international dollars per day)). * Food wages are daily laborer wages deflated by the poor person’s food price index, as described in . From Ethiopian birr they are then converted to international dollars using the 2011 purchasing power parity conversion factor for private consumption, equal to 5.57 birr to the international dollar in 2011.

Source: Authors’ analysis using ESS retail price data ([Bibr b0125][Bibr b0040]

The most Important takeaway from Fig. 2 is that, had a food security analyst only focused on food inflation in Ethiopia in the previous two decades, they would have drastically misinterpreted the magnitude of income shocks for wage earners, but also misunderstood a prolonged period (2012–2019) in which disposable wages increased dramatically, as we show below in Fig. 3, which reports the net result of the race between food inflation and wage inflation: i.e., trends in real food wages. At the start of the time period, real food wages hovered around $6–7 per day (in 2011 international dollars) from 2002 to 2006 before a 22 % fall in food wages in 2007–2008. After real wages recovered in 2009 and 2010, they again fell by 22 % in the 2011 price spike. Yet as expected from Fig. 2, we observe strong growth of real food wages after 2011, which increased by over 70 % until the end of 2019. From 2020 to 2022 the real food wage for unskilled urban workers again declined by roughly 20 percent up to June 2022. However, even by June 2022 real wages were still 30–40 percent higher than they were in the worst months of the 2008 and 2011 food crises. An important caveat, of course, is that in 2021 and 2022 wages were not reported in conflict-affected Tigray.

### 4.2 Trends in consumer food prices and food wages in Sri Lanka’s 2022 economic crisis

Since early 2022, Sri Lanka has been facing a severe economic crisis as the result of a decade of macroeconomic mismanagement in conjunction with multiple external shocks (World Bank 2022b). The crisis entailed a near depletion of foreign currency reserves, a 40 % depreciation of the rupee against major currencies from April-May 2022, exceptionally severe shortages of fuel, electricity and many imported products, and a 42 % decline in rice production in 2022 resulting from a 6-month ban on agri-chemical inputs implemented in late 2021 (FAO and WFP, 2022). A UN survey in mid-2022 estimated that 6.3 million of Sri Lanka’s 22 million population were food-insecure as a result of the crisis (FAO and WFP, 2022), despite being an upper middle-income country as recently as 2019. The World Bank (2022b) used a microsimulation approach to estimate that the $3.65/day poverty headcount doubled from 13 % in 2021 to 26 % in 2022.

Are those trends indicative of the sharp increases in food insecurity and poverty in Sri Lanka’s recent crisis?

**Fig. 4:**
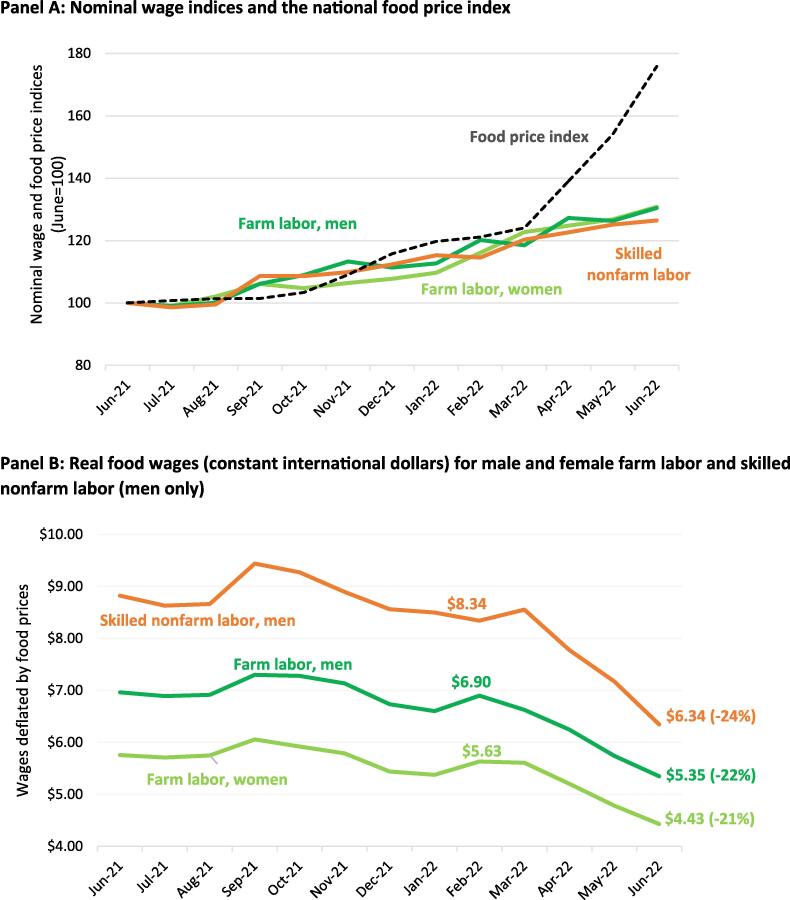
Farm and non-farm wages, food inflation and the depreciation of the rupee in Sri Lanka’s economic crisis: June 2021 to June 2022 Panel A: Nominal wage indices and the national food price index Panel B: Real food wages (constant international dollars) for male and female farm labor and skilled nonfarm labor (men only)). Food wages are wages deflated by the food price index. Real food wages are in constant 2021 international dollars.

Source: Authors’ estimates from data reported by the Central Bank of Sri Lanka (CBSL, 2022

Like many other countries, Sri Lanka collects extensive consumer food price and wage data, but the two are not linked for any type of food security monitoring, even though the Central Bank’s *Monthly Economic Bulletin* releases both series (CBSL, 2022). Panel A of Fig. 4 uses that data to first report nominal trends in the standard consumer food price index from July 2021 to June 2022 along with wages for farm labor (male and female separately), as well as non-farm skilled workers. While non-farm skilled workers may not normally be food-insecure, in the Sri Lankan context inflation was so high that even middle-income households were reported to be experiencing food insecurity from declining disposable income. In Panel A we see that nominal wages and food prices gradually trended upwards from June 2021 until March 2022, when food inflation accelerated sharply in April 2022, far outpacing wage growth. Slow growth in wages from April 2022 onwards was unsurprising given an 8 percent contraction in GDP in 2022, severe disruptions to business operations, restrictions on wage increases in the large public sector, and the especially poor performance of the agricultural sector (a major employer) due to fertilizer and fuel shortages.

Panel B shows just how quickly real food wages declined. Real wages of skilled nonfarm workers fell by 24 % in the three months between March and June 2022, while wages of male and female agricultural workers fell by 22 % and 21 %, respectively. Notable, also, is that women’s wages in the farm sector are persistently almost 20 % lower than male wages, similar to findings in rural India (Raghunathan et al., 2021).

Thus, Fig. 4 illustrates that food wages could have been a very effective and timely indicator of the declining purchasing power of relatively poor rural and urban wage earners in 2022 as the crisis unfolded. In contrast, the FAO-WFP food security and World Bank poverty analyses – though essential for a more holistic analysis of the welfare impacts of the crisis – were published 6–7 months after the onset of the inflation crisis in March 2022. An analysis of real wage trends could have much sooner revealed just how quickly purchasing power was deteriorating for men and women from different occupations in different parts of the country. Moreover, for the government itself, the marginal cost of calculating real wage trends would have been minimal since all the requisite data was already being collected.

### 4.3 Monitoring “healthy diet wages” in Myanmar’s economic and political crisis over 2021–2022

Myanmar faced a significant economic contraction due to COVID-19 in 2020, which was then immediately followed by a military coup on February 1st 2021, triggering a major financial crisis and an escalation of the country’s complex civil war. Myanmar experienced an 18 percent contraction in economic output in 2021 and continued stagnation in 2022 (World Bank, 2023), accompanied by a 50 % increase in food prices in 2022 (MAPSA, 2022a). Various phone surveys conducted over 2020–2022 reported huge increases in income-based poverty and self-reported food insecurity, as well as declining dietary diversity among adults (Boughton et al., 2023, CSO & UNDP, 2021, MAPSA, 2022c, MAPSA, 2022).

In addition to economic turbulence, the military takeover presaged a breakdown in a wide range of essential government services, including disruptions to food price monitoring and government-led welfare surveys. In this data-scarce environment, USAID funded IFPRI to conduct a high-frequency large-scale phone survey – the Myanmar Household Welfare Survey (MHWS) – four times over the course of 2022 (MAPSA, 2022, MAPSA, 2022b). MHWS collected household data on incomes, food insecurity experiences and a range of other welfare measures, and community data from food vendor surveys on food prices and daily wages of construction and agricultural workers. The food vendor survey tracked prices of a range of sentinel foods from different healthy food groups, allowing us to estimate the cost of a healthy diet as defined by food-based dietary guidelines for Myanmar (Mahrt et al., 2022, Mahrt et al., 2019).

Table 2 first reports trends in the nominal cost of healthy diets in urban and rural Myanmar, as well as nominal construction wages in urban areas and agricultural wages in rural areas, separately for men and women. With rapid inflation, the cost of a healthy diet increased by 42 % in urban Myanmar and 55 % in rural areas. Similar to Sri Lanka’s crisis, nominal wages increased but food inflation far outpaced wage inflation. In terms of levels, a daily urban construction wage in January-February 2022 could buy 4.3 healthy diet days for men and 3.3 healthy diet days for women, while an agricultural wage could buy 4.1 healthy diet days for rural men and 3.2 healthy diet days for rural women.^9^ In terms of wage declines, real wages fell by 21 % by the end of 2022 for both sexes in urban Myanmar, and by 27 % for male and 28 % for female agricultural workers in rural Myanmar.^10^

**Table 2:** Trends in nominal healthy diet costs and sector- and sex-specific wage rates for unskilled labor across four rounds of a high-frequency national phone survey in Myanmar in 2022.

	**Urban Myanmar (construction wages)**				
	**Healthy diet cost (kyat)**	**Construction wages (spatially adjusted kyat)**		**Healthy diet construction wage(wages divided by diet cost)**	
**Demographic** ≫	**Both sexes**	**Men**	**Women**	**Men**	**Women**
Jan to Feb 2022	1,686	7,277	5,633	4.3	3.3
Apr to Jun 2022	1,918	7,407	5,851	3.9	3.1
Jul to Aug 2022	1,940	7,709	6,141	4.0	3.2
Oct to Nov 2022	2,393	8,133	6,339	3.4	2.6
**Change in 2022**	**42 %**	**12 %**	**13 %**	**−21 %**	**−21 %**

	**Rural Myanmar (farm wages)**				
	**Healthy diet cost (kyat)**	**Farm wages (spatially adjusted kyat)**		**Healthy diet agricultural wage(wages divided by diet cost)**	

**Demographic** ≫	**Both sexes**	**Men**	**Women**	**Men**	**Women**
Jan to Feb 2022	1,459	5,962	4,681	4.1	3.2
Apr to Jun 2022	1,659	6,353	4,870	3.8	2.9
Jul to Aug 2022	1,797	6,412	5,009	3.6	2.8
Oct to Nov 2022	2,267	6,735	5,217	3.0	2.3
**Change in 2022**	**55 %**	**13 %**	**11 %**	**−27 %**	**−28 %**

Source: Authors’ calculations from the Myanmar Household Welfare Survey (MAPSA, 2022b). See text for details.

The availability of household data from Myanmar allows us to compare trends in real wages over 2022 to trends in real incomes of the poorest 60 % of the population within urban and rural areas, as well as trends in income-based poverty rates. Income per adult equivalent in the MHWS is derived from questions on 16 different sources of income, and poverty is defined as having an income below the 2017 poverty line, which we update to allow for inflation (MAPSA, 2022b). In urban areas the 21 % decline in healthy diet construction wages corresponds to a surprising modest 8 % decline in real incomes of the poorest 60 % of the population (Table 3), but a 19 % increase in the poverty rate. In rural areas, the 27–28 % decline in healthy diet agricultural wages corresponds to a 28 % decline in real mean incomes and a 32 % increase in the poverty rate. In both urban and rural areas, therefore, community-based real wage reductions yield very similar inferences to household poverty trends.

**Table 3:** Comparing trends in sex-specific real “healthy diet wages” to trends in real household incomes for the poorest 60% and income-based poverty rates across four rounds of a high-frequency national phone survey in rural and urban Myanmar.

	**Urban Myanmar**		
	**Healthy diet construction wage**	**Real mean incomes of the poorest 60 % (kyat)**	**Poverty rate (%)**
**Demographic** ≫	**Both sexes**	**Household**	**Household**
Jan to Feb 2022	3.83	1,555	46 %
Apr to Jun 2022	3.46	1,509	48 %
Jul to Aug 2022	3.57	1,517	49 %
Oct to Nov 2022	3.02	1,435	55 %
**Change in 2022**	**−21 %**	**−8%**	**19 %**

	**Rural Myanmar**		
	**Healthy diet farm wage**	**Real mean incomes of the poorest 60 % (kyat)**	**Poverty rate (%)**

**Demographic** ≫	**Both sexes**	**Household**	**Household**
Jan to Feb 2022	3.6	1,255	52 %
Apr to Jun 2022	3.4	995	59 %
Jul to Aug 2022	3.2	871	65 %
Oct to Nov 2022	2.6	809	68 %
**Change in 2022**	**−28 %**	**−36 %**	**32 %**

Source: Authors’ calculations from the Myanmar Household Welfare Survey (MAPSA, 2022b). See text for details. A. Median income is per adult equivalent and is deflated by a food price index. The bottom 60 % of households within urban and within rural areas is determined using spatially adjusted income.

**Fig. 5:**
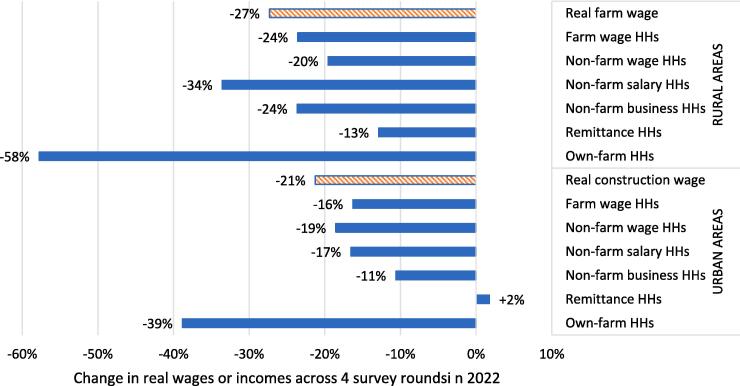
Comparing changes in real wages to changes in incomes per adult equivalent for six different household (HH) livelihood types in rural and urban areas of Myanmar across four rounds of surveys in 2022 .

Source: Authors’ calculations from the Myanmar Household Welfare Survey (MAPSA, 2022b). HHs = households

Finally, the MHWS also allows us to assess how closely real food wage movements track trends in incomes for different kinds of livelihoods, as defined by a household’s largest source of income. Fig. 5 compares changes in real food wages in 2022 (the orange striped bars) to changes in real household incomes for six different livelihoods, separately for rural and urban areas. In rural areas, the 27 % reduction in the real farm wage index is very close to the 24 % reduction in real incomes for farm wage households, a 20 % income reduction for non-farm wage households and a 24 % reduction for non-farm business households. The trends are only markedly different for non-farm salary households (−34 %), remittance-dependent households (−13 %) and own-farm households (−58 %). These results are not unexpected, however: salary-dependent are generally much less poor than other groups; remittance-dependent households in Myanmar receive income from overseas (mostly Thailand) where real wages levels and trends are essentially unaffected by Myanmar’s economic troubles; and finally, own-farm income was not expected to match wage trends because farming income is highly seasonal, with the 58 % reduction in own-farm household income in 2022 resulting from a comparison of a post-harvest time period with high rates of income from selling farm output (January-February 2022) to a pre-harvest time period with sales of farm output (October-November 2022).

In urban Myanmar we find that the 21 % reduction in the real wage index is close in magnitude to the 19 % reduction in incomes for non-farm wage households and the 16 % reduction in incomes for farm wage households. The reduction in incomes for non-farm business households is somewhat smaller in magnitude (−11 %). Finally, as with the rural sample, we observe very different income trends for remittance-dependent households (who actually see a 2 % gain in real incomes) and own farm households (−39 %) for the reasons stated above.

In summary, high-frequency household survey data from Myanmar suggest that real wage trends are not generally predictive of income trends for own-farm, salary or remittance-dependent households. In rural areas, wage trends also closely match income trends for non-farm business households, but there is a somewhat weaker correspondence in urban areas. However, wage trends very accurately reflect reductions in incomes for wage-earning households in both rural and urban Myanmar.

## 5 Conclusions and recommendations

Monitoring food security is a challenging and potentially costly exercise. Different kinds of shocks can affect different socioeconomic groups, and many kinds of shocks are difficult to predict and prepare for. The 2008 global food crisis caught LMIC governments and development partners largely by surprise; yet since then, food price volatility has become the new norm. Despite the investments development agencies have made in expanding food price monitoring in recent years, they have paid insufficient attention to monitoring wages and to measuring real food wages as a food security indicator. Nevertheless, it is important to clarify what high frequency real wage data can and cannot deliver for food security monitoring systems.

First and foremost, real wage indicators have the potential to inform agencies about potential changes in disposable income for an important segment of the poor – i.e., wage-dependent households, but potentially also households dependent on low skilled self-employment – with minimal lag-time and with temporal granularity (for example, monthly updates). Moreover, it is precisely non-farm groups who are most vulnerable to food price shocks, and often also more vulnerable to macroeconomic shocks that affect nominal wages and employment. In contrast, agricultural households are often somewhat protected from macroeconomic shocks but are highly vulnerable to agro-climatic shocks. Hence real wage indicators are a perfect complement to more agriculture-focused food security indicators.

Second, healthy diet wages might also be used as a more nutrition-oriented measure. Analogous to methods developed in the *Fill the Nutrient Gap* approach,^11^ one could develop proxies for the affordability of healthy diets for wage-earning households to identify meaningful thresholds at which wage rates make healthy diets unaffordable. Hence, healthy diet wages could provide a high-frequency analog to the infrequently updated “affordability of a healthy diet” metric now reported in the UN’s annual *State of Food Insecurity and Nutrition* reports (FAO, et al., 2022).

Third, while “community” level wage and price data cannot directly be used for social protection targeting assessments, there are nevertheless untapped opportunities for more granular real wage measurement to yield information on the extent to which different occupations and economic activities are affected by shocks, and how these impacts differ by gender. In India a vast array of wage data are collected in both rural and urban areas for a wide variety of economic activities, with disaggregation by gender (Raghunathan et al., 2021).^12^ The same is true in Sri Lanka. Yet such data are simply not used for food security analysis, even in the current context of exceptionally high inflation rates. Nevertheless, the potential for real wage data to provide timely information for social protection programming is self-evident.

Fourth, food wages can be useful for assessing the appropriate magnitude of social protection transfers for non-farm households, especially in the context of rapidly changing food prices and/or wages. In rural India Raghunathan et al. (2021) were able to compare the cost of a healthy diet to the state-specific minimum wages of one of India’s most important social protection programs, and show that the value of social protection wages declined by 25 % from 2006 to 2011 as food prices increased.^13^ Data on healthy diet costs and wages can also be used to explore how the adequacy of social protection varies by season, since food prices are highly seasonal in many LMICs, especially in rural areas.

Section 3 already outlines the limitations of real wage data for food security measurement, and we only briefly reiterate them here. Apart from having limited relevance to farming-dependent households and rural areas where in-kind wages are still common, real wage measurement also needs to consider underemployment, or average hours worked. In India, average hours worked is often measured with wage rates, which shows that it may indeed be possible to estimate average wage earnings rather than wage rates.

A final challenge Is that wage data may be collected but not disseminated at all, not disseminated quickly enough for food security monitoring, or not disseminated in a granular fashion for specific subnational regions, activities and genders.^14^ Unfortunately, lack of public dissemination of wage data is also matched by lack of dissemination of food price data in many countries (Bai, et al., 2021).

However, despite this unfortunate data dissemination challenge, we envision several practical opportunities to develop the measurement and dissemination of different types of real wage indicators.

First, diplomatic approaches could be used to promote greater inter-agency cooperation on data-sharing. The WFP already works closely with national governments to access consumer food price data, and similar diplomatic efforts could potentially “unlock” wage data in a timelier fashion. In some cases, more cooperation is needed between different government agencies since multiple agencies collect different price and wage data, and nutrition agencies could also play a role in developing healthy diet cost metrics. In Ethiopia, a collaboration between The Ethiopian Public Health Institute (EPHI), the Ethiopian Statistical Services (ESS), IFPRI and Tufts University – with support from The Bill and Melinda Gates Foundation – have started producing regular bulletins on the cost and affordability of healthy diets using the same ESS monthly price and wage data we analyzed above (Alemayehu et al., 2023).

Second, technological advances are rapidly changing our capacity to collect data on prices and wages cost-effectively. The Myanmar case study shows conduct a national high-frequency food vendor survey by phone in extremely challenging economic and political circumstances for around 20,000 USD per round (MAPSA, 2022a). Phone surveys clearly hold great promise for high frequency food security monitoring.

A third approach is to encourage agencies that already collect food price data to also collect wage data. In Sri Lanka IFPRI has collaborated with the Hector Kobbekaduwa Agrarian Research and Training Institute (HARTI) to add wage quotations to their already extensive food price surveys for the purposes of monitoring healthy diet wages/affordability.

Finally, in all these instances there are opportunities to develop integrated price and wage dashboards to disseminate real wage data in a timely fashion.

On the basis of the arguments above, we strongly urge international aid agencies involved in food price monitoring – particularly the WFP, FAO, USAID, ILO, CGIAR and national governments – to either collect wage data in their own surveys, or to seek out existing wage and price data that is collected but not yet used for food security analysis; in short, to pluck this very low hanging fruit. The scientific community and UN agencies have made great progress on defining what we mean by “sufficient … nutritious food”, but the challenge of monitoring the affordability of nutritious food for “all people, at all times” (FAO, 1996) is not yet met. Measuring the wages of the poor constitutes a large but inexpensive step towards meeting that challenge.

## CRediT authorship contribution statement

**Derek Headey:** Conceptualization, Data curation, Formal analysis, Funding acquisition, Investigation, Methodology, Writing – original draft, Writing – review & editing. **Fantu Bachewe:** Formal analysis. **Quinn Marshall:** Conceptualization, Funding acquisition, Methodology, Writing – original draft. **Kalyani Raghunathan:** Conceptualization, Methodology, Writing – original draft, Writing – review & editing. **Kristi Mahrt:** Conceptualization, Data curation, Formal analysis, Investigation, Methodology, Writing – original draft.

## Footnotes

1 For the FAO, seehttps://www.fao.org/giews/en/, for USAID see https://fews.net/fews-data/337, and for WFP see https://www.wfp.org/publications/market-monitor). In many instances these food price monitoring dashboards report food prices collected by other agencies, but the WFP often collects its own food price data through surveys conducted primarily in its programming areas, while several UN organizations make use of national consumer price surveys to access data on item-level food prices, even though the main purpose of such surveys is to estimate both food and aggregate inflation. These international systems also rely on improved food price monitoring by developing countries themselves.

2 The annual SOFI reports in 2020, 2021 and 2022 are derived through extrapolations from food price data collected in the 2017 ICP round, while income and expenditure data likewise come from national household surveys that are similarly infrequent. Moreover, SOFI healthy diet affordability estimates do not contain information at the subnational level, nor gender-disaggregated statistics.

3 See https://www.fao.org/3/nf197en/nf197en.pdf.

4 Contemporary economic historians also analyze food wages to track historical changes in real incomes (Allen, 2001; Bassino and Ma, 2006; Bhattacharya and Rama Deb, 1977; Broadberry and Gupta, 2006).

5 In Myanmar, for example, 2020 saw price increases in poultry products and vegetables due to COVID-19 related transport disruptions, 2021 saw a large increase in imported palm oil prices, 2022 saw general inflation in most food products due to the country’s currency depreciation and other economic disruptions, and 2023 saw rice price increases due to rising international prices (Boughton et al., 2021; Boughton et al., 2023).

6 Or, alternatively, the cost of grains such as rice, wheat or maize, which may be an appropriate but very basic food security indicator in some contexts.

7 Likewise, one would ideally want to assess affordability after accounting for non-food expenditure needs, which could be estimated either from existing national non-food poverty lines, or alternatively through context-specific basic needs estimation techniques such as those proposed by Allen (2017) and extended by Headey et al. (2023). Whether one uses it in high frequency real wage monitoring depends on the availability of non-food prices or price indices.

8 Countries included were Ethiopia, Malawi, Niger, Nigeria, Tanzania, and Uganda.

9 Obviously, factoring in the need to buy healthy diets for non-working dependents would reduce disposable income from food wages, as would factoring in non-food expenditure requirements, though we do not engage in that exercise here.

10 Note that we also estimated wage declines using a more conventional food price index. These declines were similar to those in Table 2 for rural areas but somewhat steeper in urban areas, being 26% for male construction workers and 25% for female construction workers.

11 See https://www.wfp.org/publications/2020-fill-nutrient-gap for a description of the various uses of the *Fill the Nutrition Gap* approach to costing nutrient-adequate diets adopted by the WFP.

12 In India, Raghunathan et al. (2021) obtained wage data for 18 different agricultural activities disaggregated by gender and showed that men’s wages were typically 50% higher than women’s wages. In Sri Lanka, wages are collected for urban activities, crop activities and estate sector activities (tea, rubber), which is important given the much poorer economic and nutrition conditions of the estate sector population. It may also be advisable to combine wage rates for a range of different activities into a single index or series of indices (e.g., an agricultural wage rate index).

13 Raghunathan et al. (2021) examine the food purchasing power of the National Rural Employment Guarantee Act (NREGA), which entitles every rural household to 100 days of unskilled labor at the state minimum wage. They showed that while NREGA wages were generally sufficient to purchase four days of a healthy diet in 2006 when the program started, the rising real cost of a healthy diet resulted in NREGA wages only being sufficient to purchase three days of a healthy diet by 2011; a 25% decline in the purchasing power of social protection wages.

14 India collects an astonishingly rich array of wage data, but much of it is not in the public domain, and its release is not timely. At the time of writing (December 2023), agricultural wage data from the Ministry of Agriculture and Farmers Welfare was only updated to 2020–21; i.e. a 2 year lag.
