# Supplementary material for: Food prices and the wages of the poor: A cost-effective addition to high-frequency food security monitoring
Source: Food Policy. 2024 May;125:102630. doi: 10.1016/j.foodpol.2024.102630 (PMC11190886; doi:10.1016/j.foodpol.2024.102630)
Supplement: Supplementary Data 1 [file mmc1.docx]

**ONLINE ONLY APPENDICES**

**Appendix Table A1. The share of women paid in-kind or both cash and in-kind in rural and urban areas in 62 countries with Demographic Health Surveys**

|  | **Rural Areas** | | **Urban areas** | |
| --- | --- | --- | --- | --- |
|  | **wage in-kind** | **cash & in-kind** | **wage in-kind** | **cash & in-kind** |
| ***Eastern Europe & Central Asia*** |  |  |  |  |
| Albania | 10% | 11% | 11% | 1% |
| Armenia | 29% | 21% | 21% | 2% |
| Azerbaijan | 12% | 13% | 13% | 2% |
| Kyrgyz Republic | 4% | 1% | 1% | 3% |
| Moldova | 14% | 10% | 10% | 1% |
| Tajikistan | 12% | 5% | 5% | 5% |
|  |  |  |  |  |
| ***Latin America & Caribbean*** |  |  |  |  |
| Bolivia | 15% | 10% | 10% | 3% |
| Colombia | 8% | 2% | 2% | 6% |
| Dominican Rep. | 8% | 0% | 0% | 7% |
| Guatemala | 4% | 2% | 2% | 3% |
| Guyana | 5% | 2% | 2% | 0% |
| Haiti | 58% | 0% | 0% | 45% |
| Honduras | 12% | 2% | 2% | 6% |
| Nicaragua | 3% | 6% | 6% | 2% |
| Peru | 18% | 6% | 6% | 10% |
|  |  |  |  |  |
| ***Middle East & North Africa*** |  |  |  |  |
| Egypt | 3% | 1% | 1% | 1% |
| Yemen | 11% | 15% | 15% | 4% |
|  |  |  |  |  |
| ***South & South-East Asia*** |  |  |  |  |
| Bangladesh | 10% | 1% | 1% | 7% |
| Cambodia | 8% | 5% | 5% | 6% |
| India | 13% | 4% | 4% | 6% |
| Maldives | 2% | 1% | 1% | 1% |
| Myanmar | 9% | 5% | 5% | 1% |
| Nepal | 30% | 12% | 12% | 12% |
| Pakistan | 6% | 5% | 5% | 0% |
| Timor-Leste | 13% | 3% | 3% | 6% |
|  |  |  |  |  |
| ***Sub-Saharan Africa*** |  |  |  |  |
| Angola | 36% | 13% | 13% | 5% |
| Benin | 20% | 2% | 2% | 12% |
| Burkina Faso | 29% | 25% | 25% | 9% |
| Burundi | 50% | 28% | 28% | 16% |
| Cameroon | 59% | 6% | 6% | 23% |
| Chad | 22% | 3% | 3% | 13% |
| Comoros | 21% | 8% | 8% | 11% |
| Congo, Rep. | 23% | 3% | 3% | 4% |
| Congo, DRC | 60% | 16% | 16% | 20% |
| Ethiopia | 43% | 14% | 14% | 21% |
| Gabon | 16% | 3% | 3% | 3% |
| Gambia | 43% | 4% | 4% | 13% |
| Ghana | 26% | 8% | 8% | 19% |
| Guinea | 15% | 9% | 9% | 4% |
| Kenya | 21% | 8% | 8% | 13% |
| Lesotho | 3% | 5% | 5% | 0% |
| Liberia | 18% | 7% | 7% | 10% |
| Madagascar | 45% | 38% | 38% | 11% |
| Malawi | 25% | 10% | 10% | 3% |
| Mali | 17% | 5% | 5% | 2% |
| Mozambique | 21% | 47% | 47% | 7% |
| Namibia | 12% | 1% | 1% | 1% |
| Niger | 13% | 3% | 3% | 4% |
| Nigeria | 16% | 1% | 1% | 9% |
| Rwanda | 59% | 18% | 18% | 24% |
| Sao Tome and Principe | 20% | 4% | 4% | 19% |
| Senegal | 20% | 5% | 5% | 0% |
| Sierra Leone | 23% | 5% | 5% | 6% |
| South Africa | 1% | 2% | 2% | 2% |
| Swaziland | 4% | 2% | 2% | 3% |
| Tanzania | 22% | 3% | 3% | 5% |
| Togo | 16% | 10% | 10% | 1% |
| Uganda | 40% | 5% | 5% | 16% |
| Zambia | 37% | 3% | 3% | 7% |
| Zimbabwe | 27% | 4% | 4% | 7% |

Source: Results estiamted from the most recent survey round in each country with a Demographic Health Surveys (ICF International, 2022).

**Appendix Table A2. Forms of payment for working women in 35 countries in sub-Saharan Africa**

|  |  | **Form of payment** | | | |
| --- | --- | --- | --- | --- | --- |
|  |  | **Not paid** | **Cash-only** | **In-kind only** | **Both** |
|  |  |  |  |  |  |
| **Primary occupation** | **Observations** |  |  |  |  |
| agriculture | 135,559 | 47% | 15% | 25% | 14% |
| sales | 73,677 | 8% | 82% | 9% | 1% |
| skilled manual | 16,591 | 9% | 81% | 9% | 1% |
| unskilled manual | 10,860 | 14% | 76% | 8% | 2% |
| domestic work | 4,045 | 7% | 84% | 8% | 1% |
| services | 14,514 | 10% | 83% | 7% | 1% |
| professional | 10,643 | 5% | 90% | 3% | 1% |
| clerical | 2,361 | 3% | 95% | 2% | 0% |
| other | 5,132 | 26% | 33% | 26% | 14% |

Source: Demographic Health Surveys (ICF International, 2022). “Agriculture” includes women who report working on their own-farm as their main occupation (which explains the 47% reporting that they are not paid any wages) as well as women who report working on other farms.
